# Enzymes and Enzyme Inhibitors—Applications in Medicine and Diagnosis

**DOI:** 10.3390/ijms24065245

**Published:** 2023-03-09

**Authors:** Athina Geronikaki, Phaedra T. Eleutheriou

**Affiliations:** 1 School of Pharmacy, Aristotle University of Thessaloniki, 54124 Thessaloniki, Greece; 2 Clinical Chemistry-Biochemistry, International Hellenic University, 57400 Thessalonik, Greece

This is the first part of a Special Issue on enzymes and enzymes inhibitors and their applications in medicine and diagnosis.

The first paper focused on the maintenance of MBCOMT’s stability in situations where the protein needs to be stored for long periods of time. Membrane-bound catechol-O-methyltransferase (MBCOMT) is responsible for the main pathway of catechol neurotransmitter deactivation. This enzyme is linked to several types of human dementia, and new, potent, nontoxic inhibitors have been developed for Parkinson’s disease treatment. However, the enzyme’s instability represents a major obstacle to new drug development, as it tends to lose its biological activity quickly. Ionic liquids, as has recently been shown, can help to preserve protein stability and folding and prevent protein aggregation due to their diverse ion combinations. The addition of additives to enzyme buffer, such as cysteine, glycerol, and trehalose, has shown promising results in minimizing MBCOMT damage and enhancing its stability. The results revealed that the buffer used by the authors not only led to the maintenance of hMBCOMT activity for up to 32.4 h, enabling storage at −80 °C, but also increased the biological activity by up to approximately 40% compared to its original level [1].

The goal of the second paper was the assessment of hMBCOMT’s stability in situations where the protein needs to be stored for long periods of time. The authors tested several healthy control tear samples for the validation of the assay, and then 20 tear samples from patients diagnosed with cataracts, glaucoma, allergy, dry eye, and meibomian gland dysfunction were studied. The obtained results confirmed the reliability of the AbMAs test for the quantification of the MMP-9 concentration in human tear samples. Thus, the authors concluded that the use of biomarker detection technologies is also advantageous for the evaluation of the prognosis and render the work of the ophthalmologist easier, thus leading to greater improvements in patients’ health [2].

The third paper explores development of the mpelanin-concentrating hormone receptor 1 (MCHR1) antagonist, which is useful for treating obesity. Taking into account the fact that its binding site is similar to the human (hERG) channel and that most drugs developed as MCHR1 antagonists have failed in clinical development because of the cardiotoxicity caused by hERG, machine-learning-based prediction models can be useful for overcoming these difficulties. Taking this into account, the authors attempted to discover novel MCHR1 antagonists without cardiotoxicity using DNN-based machine learning models and to identify new indications by analyzing gene expression. As a result, the authors identified a KRX-104130 MCHR1 antagonist without cardiotoxicity. Moreover, it was found that by using a transcriptome-based drug repositioning approach, it becomes possible to identify new indications for this antagonist. Thus, the authors showed that KRX-104130 increased the expression of low-density lipoprotein receptor (LDLR), which is responsible for the reduction in the cholesterol levels. Furthermore, it was mentioned that this antagonist demonstrated a protective effect by reducing the degree of hepatic lipid accumulation, liver injury and histopathological changes. Moreover, using GO/KEGG analysis, it was found that KRX-104130 is insulin-resistant [3].

The next paper concerns the search of inhibitors of hexokinase 2 (XK2), an enzyme of the sugar kinase family with dual roles in glucose metabolism and the mediation of cancer cell apoptosis. It was found that the inhibition of this enzyme improves the efficacy of cancer drugs. Taking into account the previous reports regarding the potent HK2 inhibitory activity of benitrobenrazide (BNBZ), the aim of this work was to synthesize the parent BNBZ and its three dihydroxy derivatives in order to carry out additional physicochemical and biological studies. In order to test the hypothesis regarding the effects of the number and position of hydroxyl groups on HK2 inhibitory activity, the authors performed several in vitro studies using two human liver cancer cell lines, HepG2 and HUH7. This study revealed that modifications to the structures of new BNBZ significantly affected their activities. It was found that these compounds have a tendency to exhibit toxic effects and cause aggregation. In addition, it appears that they contribute to DNA damage, increase ROS production, and disrupt of cell progression. In conclusion, the authors stated that the obtained results are preliminary in nature, and more investigations are required in order to elucidate the exact mechanism of action and all the side effects [4].

The fifth paper concerns a novel Aurora A kinase inhibitor, fangchinoline, against ovarian cancer. Aurora kinase is a serine/threonine kinase regulator controlling multiple events during cell cycle progression. Since this kinase plays important roles in promoting the proliferation of cells and inhibiting cell death in cancer, it appears to be a good target for cancer therapy. In this paper, a natural compound with higher docking scores than the known Aurora A ligand was identified by structure-based virtual screening. In this screening, fangchinoline was also included, since its anticancer activity is known but its effect on ovarian cancer is not. Since the usual therapy for ovarian cancer is cisplatin, fangchinoline combined with cisplatin was used, and it was found that this combination enhanced the cisplatin–DNA adduct levels, and the combination index demonstrated a synergistic effect. In vivo experiments on mice revealed that fangchinoline supported and enhanced the anticancer activity of cisplatin against ovarian cancer [5].

The next paper explores the role of urate oxidase, which initiates the uric acid degradation pathways and is widely used in protein drug development for gout therapy and serum uric acid diagnosis. The main goal of the authors was the characterization of a thermostable urate oxidase from *Deinococcus radiodurans* (DrUox) with a high catalytic efficiency and thermal stability, with the aim of using it for medical applications. The authors described a thermostable urate oxidase from *D. radiodurans* in terms of its biochemical aspects, with the specific activity of DrUox being 38.06 U mg^−1^ higher than that of the reported urate oxidases. Furthermore, it retains almost 100% activity for 24 h, compared to urate oxidases from other sources, which retain their activity for only 1 h after incubation. Thus, it can be concluded that a thermostable urate oxidase with a high catalytic efficacy and thermal stability may be considered as a potential target for drug development [6].

The seventh paper is an extended review of the development of new treatments for COVID-19 using dual-action antiviral/anti-inflammatory small molecules and physiologically based pharmacokinetic modeling. The authors refer to the molecular targets of SAR Cov-2, known targets for anti-inflammatory agents against COVID-19, antiviral drugs used for the treatment of COVID-19, dual-acting compounds considered as potent drugs that could be used to cure COVD-19, and the role of computer-aided drug design and its application in cases of COVID-19. They present a review regarding structure-based and ligand-based drug design, as well as the PBPK modeling of anti-COVID-19 small molecules. In conclusion, the authors reported that small molecules possessing dual antiviral and anti-inflammatory (AAI) activities may help to simplify therapy, prove to be more effective, prevent relapse, and reduce long-term COVID-19 complications. Furthermore, dual AAI molecules may be especially useful in resource-challenged countries, where access to new anti-inflammatory agents and monoclonal antibodies may be limited. Additionally, they pointed out the roles of computational methods, as well as physiologically based pharmacokinetic (PBPK) modeling, in the discovery and development of new drug candidates against COVID-19 [7].

The last paper in this Special Issue is a review of the characteristics of food-protein-derived antidiabetic bioactive peptides. The authors provide an overview of the DPP-IV, PTP-1B, and α-glucosidase inhibitors and updated information on the methods used for the discovery of DPP-IV inhibitory peptides released from food protein hydrolysate, since the inhibition of all these enzymes is promising for the treatment of diabetes type 2. The authors highlight the status of the dietary proteins as resources of antidiabetic peptides, PTP1B, and a-glucosidase inhibitory peptides, with their roles in diabetes type 2 and its mechanism of action, as well as DPP-IV inhibitory peptides. The greatest attention is given to a discussion regarding DPP-IV inhibitory peptides with respect to peptide sequestration; the in silico prediction of potential DPP-IV inhibitory peptides, their characteristics, and inhibition modes; and stability quantification through molecular docking. Additionally, the authors mention the effects of food-derived DPP-IV inhibitory peptides observed in animal and clinical studies conducted in vivo.

In conclusion, the authors demonstrated that peptides have promising biological effects, are safe and, in general, enable low cost-effective research. However, despite the fact that food-derived peptides have many advantages, their use as functional foods or pharmaceutical drugs requires careful and sufficient examination in combination with clinical added [8].
